# Diagnostic performance of different anthropometric indices among Iranian adolescents for intima media thickness in early adulthood: A prospective study and literature review

**DOI:** 10.3389/fnut.2023.1098010

**Published:** 2023-02-28

**Authors:** Golaleh Asghari, Ali Nikparast, Maryam Mahdavi, Pooneh Dehghan, Majid Valizadeh, Farhad Hosseinpanah, Fereidoun Azizi, Farzad Hadaegh

**Affiliations:** ^1^Nutrition and Endocrine Research Center, Research Institute for Endocrine Sciences, Shahid Beheshti University of Medical Sciences, Tehran, Iran; ^2^Department of Clinical Nutrition and Dietetics, Faculty of Nutrition Sciences and Food Technology, Shahid Beheshti University of Medical Sciences, Tehran, Iran; ^3^Obesity Research Center, Research Institute for Endocrine Sciences, Shahid Beheshti University of Medical Sciences, Tehran, Iran; ^4^Department of Imaging, Taleghani Hospital, Shahid Beheshti University of Medical Sciences, Tehran, Iran; ^5^Endocrine Research Center, Research Institute for Endocrine Sciences, Shahid Beheshti University of Medical Sciences, Tehran, Iran; ^6^Prevention of Metabolic Disorders Research Center, Research Institute for Endocrine Sciences, Shahid Beheshti University of Medical Sciences, Tehran, Iran

**Keywords:** obesity, child, adolescent, carotid intima-media thickness, atherosclerosis, adulthood, longitudinal cohort study

## Abstract

**Background:**

There is debate regarding which anthropometric indices is the most appropriate predictor of cardiovascular disease (CVD) among adolescents. The purpose of this study was to investigate the association of body mass index (BMI), waist circumference (WC), waist-to-hip ratio (WHR), and waist-to-height ratio (WHtR) in adolescents with high carotid intima-media thickness (cIMT) in early adulthood, as the surrogate marker of CVD in a cohort study.

**Methods:**

A total of 875 Iranian adolescents (female = 421) aged 10–17 years old were entered the study. The cIMT was measured in early adulthood (20–38 years old) after 18.2 (median) years of follow-up and defined as > 90th percentile for sex and age groups. The gender specific association between a 1-SD increase in each anthropometric measures with high cIMT was examined using multivariate logistic regression analysis adjusted for age, smoking, family history of CVD, systolic blood pressure, total cholesterol, and fasting blood sugar. In the multivariable analysis, the interaction between sex and age groups with anthropometric measures were significant (all *p*-values < 0.05).

**Results:**

Among males, all anthropometric measures including BMI, WC, WHR, and WHtR were associated with high cIMT; the corresponding odds ratios were 1.43 (1.05–1.94), 1.63 (1.22–2.19), 1.33 (1.03–1.71), and 1.41 (1.07–1.87), respectively. However, after considering the related adulthood anthropometric measurements, the association remained significant for WC 1.48 (1.04–2.10) and WHR [1.28 (0.99–1.66), *P* = 0.06]. Moreover, among early adolescent boys aged 10–14 years, all of the anthropometric measures were significantly associated with high cIMT in the multivariate analysis that included the related adulthood anthropometric measures. The area under the curve (AUC) for the anthropometric measurements among males ranged from 0.576 for WHtR up to 0.632 for WC, without any superiority between them. Among females, only in linear regression analysis, a significant association were found between the higher value of WC and WHtR with cIMT measurement in adulthood; however, the risk reached to null after considering adult anthropometric measures.

**Conclusion:**

General and central obesity measures were significantly associated with high cIMT only among Iranian male adolescents, the relationship that were more prominent among pre-pubertal males.

## Introduction

Cardiovascular disease (CVD) is the leading cause of morbidity and mortality, accounting for nearly 17.9 million deaths annually and approximately 32% of all deaths worldwide (1). Despite the decline in CVD mortality in many industrialized countries, at least three out of four premature deaths were occur in low- and middle-income countries due to CVD and other non-communicable diseases (1). Atherosclerosis, as a lifelong disease, is known as one of the major causes of CVD. Although the clinical manifestations of atherosclerosis usually appear after midlife, the disease process has a long silent stage that is initiated as early as childhood (2). Therefore, early identification of individuals with subclinical atherosclerosis is crucial to implementing primary preventative strategies to reduce CVD risk in adulthood (3).

Several studies have shown a link between childhood cardiometabolic risk factors, including elevated blood pressure, glucose intolerance, abnormal blood lipids, and obesity, with the development of subclinical and clinical CVD in adulthood (4–7). Carotid intima-media thickness (cIMT), as measured by ultrasonography, is a validated, sensitive, and reproducible technique for detecting and quantifying subclinical atherosclerosis in asymptomatic individuals that corresponds with the development of coronary artery disease and has been identified as a predictor of future cardiovascular events as well as all-cause mortality (8). According to the meta-analysis conducted in 2013, one standard deviation increase in cIMT increased the risk of myocardial infarction and stroke by 26 and 31%, respectively (9). Furthermore, increased cIMT was found to be more strongly associated with mortality and cardiovascular endpoints in individuals younger than 50 years (10).

In spite of the efforts of the World Health Organization to achieve the goal of “halting the increase in obesity prevalence by 2025,” none of the countries have met this objective, and it is predicted that one out of eight children and adolescents in the world will be obese by 2030 (11). According to a systematic review investigating childhood risk factors and adulthood CVDs risk, 19 of the 20 studies have reported a significant association between childhood adiposity and thicker cIMT in adulthood (4). In a study published by Hosseinpanah et al. childhood body mass index (BMI) was found to be a strong predictor of cIMT in early adulthood (12). Furthermore, another study indicated that one standard deviation increase in adolescent BMI corresponded to a 2.3 μm increase in cIMT in ages between 27 and 30 years (13). Although BMI is the most commonly used index for the evaluation of general obesity, it does not consider body fat distribution and abdominal fat mass, which is particularly associated with increased CVD risk (14).

According to our knowledge, this study represents the first of its kind to examine the association between childhood central obesity measurements, including waist circumference (WC), waist-to-hip ratios (WHR), and waist-to-height ratio (WHtR), in addition to body mass index (BMI) and risk of high cIMT in early adulthood. Therefore, in this prospective study, we investigate the association between childhood adolescent anthropometric indices, including BMI, WC, WHR, and WHtR, with high cIMT in early adulthood during about two decades of follow-up.

## Materials and methods

### Study population

The current longitudinal study was performed within the framework of the Tehran Lipid and Glucose Study (TLGS). The TLGS is an ongoing large scale population-based prospective study aiming to investigate and prevent non-communicable diseases (NCDs) risk factors and promote a healthy lifestyle to reduce these risk factors (15). The study participants were followed up every 3 years according to standard methods to monitor their demographics, lifestyle, biochemical profile, clinical information, and anthropometric indices. The baseline survey was a cross-sectional study including 15005 participants aged ≥ 3 years old who were selected using a multistage random sampling method in district 13 of Tehran, conducted from 1999 to 2001. Survey 2 (2002–2005), survey 3 (2006–2008), survey 4 (2009–2011), survey 5 (2012–2015), and survey 6 (2016–2019) were prospective follow-up surveys; this cohort is still being followed up.

At the recruitment, we collected data on 2,660 participants aged between 10 and 17 years old. Exclusions included those with prevalent cancer (*n* = 1), corticosteroid users (*n* = 8), and those without follow up till survey 5 or 6 (*n* = 981), leaving 1,670 participants. Out of these individuals, following phone contact with them, 711 individuals did not attend the medical center or express their willingness to participate in the cIMT measurement. Of the remaining individuals who measured the cIMT (*n* = 959), (missing data of cIMT, *n* = 5) the data of valid IMT were available on 954 cases. Finally, after excluding those with prevalent CVD (*n* = 8), and those with missing data on covariates at the end of study (*n* = 71), 875 individuals (Male = 454) were entered into our data analysis. The study protocol has been approved by the institutional ethics committee of the Research Institute for Endocrine Sciences, affiliated to the Shahid Beheshti University of Medical Sciences, and was conducted in accordance with the principles of the Declaration of Helsinki. At the beginning of this study, written informed consent was obtained from all parents or legal guardians of participants under 18. As well, all participants ≥ 18 years signed a written informed consent form before participating in the investigations.

### Anthropometric and laboratory assessment

A detailed description of the TLGS protocol and laboratory procedures has been provided elsewhere (15). Briefly, obtaining demographic information and taking anthropometric measurements were performed by trained examiners in accordance with standardized protocols. Weight was measured while the participants were minimally dressed and without shoes, using a digital scale (Seca 707, Hanover, MD, USA) and recorded to the nearest 100 g. Height was measured while standing, without shoes, with shoulders held in a normal position, using a tape meter. Waist circumference (WC) was measured at the midpoint level (umbilicus level) while the subjects were in a standing position using an un-stretched tape meter, without any pressure on the body surface, and hip circumference (HC) was measured over light clothing at the widest girth of the hip using a tape meter. These measurements were recorded to the nearest 0.1 cm. Body mass index [BMI = weight (kg)/square of height (m^2^)], waist-to-hip ratio [WHR = waist circumference (cm)/hip circumference (cm)], and waist-to-height ratio [WHtR = waist circumference (cm)/height circumference (cm)] were calculated. Systolic and diastolic blood pressures were measured by a qualified physician in a seated position following the participant’s rest for 15 min, using a standard mercury sphygmomanometer (calibrated by the Iranian Institute of Standards and Industrial Researches) with the cuff placed on the right brachial at the heart level. Blood pressure was measured twice, at least a 30-s interval, and then the average of two measurements was recorded as the participant’s blood pressure. After 12–14 h of overnight fasting, blood samples were taken from all subjects and centrifuged within 30–45 min of collection. All blood analyses were conducted at the TLGS research laboratory on the day of blood collection using commercially available laboratory kits (Pars Azmoon Inc., Tehran, Iran) adapted to a Selectra 2 auto analyzer (Vital Scientific, Spankeren, Netherlands). Fasting plasma glucose (FPG) was assayed using the glucose oxidase based on the enzymatic colorimetric technique. Inter- and intra-assay coefficients of variation were both 2.2% for serum glucose. Serum total cholesterol (TC) and triglycerides (TGs) were also measured by enzymatic calorimetric methods with cholesterol esterase and cholesterol oxidase, and glycerol phosphate oxidase, respectively. Inter- and intra-assay coefficients of variation were 2 and 0.5% for TC and 1.6 and 0.6% for TGs, respectively. High-density lipoprotein cholesterol (HDL-C) was assayed following the precipitation of the apolipoprotein B-containing lipoproteins with phosphotungstic acid. The low-density lipoprotein Cholesterol (LDL-C) was calculated by the Friedewald formula based on the serum TC, TG, and HDL-C concentrations expressed in mg/dl if the serum TG concentrations were less than 400 mg/dL (16).

### Carotid intima-media thickness assessment

Two qualified radiologists, who were blinded to the study participant’s details, measured the intima-media thickness of extra carinal carotid arteries based on B-mode ultrasound imaging techniques using a linear 7.5–10 MHz probe (Samsung Medison SonoAce R3 ultrasound machine). The measurement was done on both right and left carotids in the supine position, with the neck extended and slightly rotated to the opposite side as a first step, transverse carotid scan was conducted to evaluate the subject’s anatomy, locate atherosclerotic plaques (if present), and determine the site of maximal wall thickening in the near or far wall. Afterward, longitudinal scans with different angles were obtained from the artery. In order to obtain optimal gray scale imaging of the carotid artery, measurements on plaque-free arterial segments were implemented according to optimal B-mode imaging criteria, which is defined as Clear visualization of far wall arterial interfaces with completely anechoic luminal content. A scan depth adjustment was attempted in order to bring the arterial lumen to the center of the image while setting the focal zone at the level of the arterial lumen. Generally, IMT was regarded as a hypo-echoic band between the arterial wall’s echogenic intimal and adventitial surfaces. The cIMT was calculated by measuring three locations on either side of the distal segment of the common carotid artery between the leading edge of the first and second echogenic lines. The average measurements were taken as the final measurement on each side. IMT measurements were sporadically conducted on the distal segments of both sides of the internal carotid artery and carotid bulb in subjects who met the optimal technique and image criteria. In the present study, as measurements taken along the left common carotid artery (LCCA) displayed less inter- and intra-observer variation and were more in line with laboratory test results, we used left common carotid artery far wall measurements (LCCA) for defining high cIMT. In order to test the rate of reliability agreement, cIMT was measured by both radiologists in a subsample of 30 participants, consisting of 66.7% females with the mean age and BMI of 41.7 ± 10.7 years and 24.4 ± 5.5 kg/m^2^, respectively. The degree of agreement between the two radiologists with regard to the cIMT measurements was evaluated using the inter-class correlation coefficient (ICC) and their 95% confidence intervals based on the two-way mixed-effects model. According to the ICC analysis, the ICC values and 95% CI were 0.79 and 0.55–0.90, respectively. In general, the ICC ranges between 0 and 1, where values between 0.75 and 0.9 indicate good reliability (17).

### Definitions

Family history of premature cardiovascular diseases was defined as a prior history of myocardial infarction, stroke, or sudden cardiac death in a male first-degree relative or father grandparent < 55 years old and in a female first-degree relative or mother grandparents < 65 years old. A high cIMT (as a surrogate for subclinical atherosclerosis) was defined as CIMT greater than the 90th percentile values specific for sex and age group (18). Smoking habits were classified into two groups: (a) current smokers, (b) past/never smokers.

### Statistical analysis

Baseline characteristics of subjects were shown as a mean ± standard deviation (SD) and median and inter-quartile range (IQR) 25–75 for normally distributed continuous variables and skewed-distributed continuous variables, respectively. Categorical variables of baseline characteristics were presented as frequency (percentages). Characteristics of participants at baseline and the end of follow-up between sex as well as respondent and non-respondent (those with missing data on covariates, those who did not have any follow up, and those who did not participate in the cIMT measurement) were compared using an independent sample *t*-test, Mann–Whitney U test, and Chi-square test as appropriate. In order to examine the effect modification of sex and age groups on the impact of each anthropometric measures for high cIMT, logistic regression analyses were conducted. In the multivariable models, the interaction between sex and age groups with BMI, WC, and WHtR were significant (all *p*-values < 0.05). Therefore, all analysis were conducted separately for boys and girls in two age groups.

The association between different adolescent anthropometric indices and high cIMT in early adulthood were evaluated by calculating the multiple-adjusted odds ratios (ORs) using logistic regression analysis. ORs and 95% confidence intervals (CIs) were evaluated per 1-SD of BMI, WC, WHR, and WHtR as continuous variables for the total boys and girls as well as in age-stratified groups in each gender. Multiple linear regression was also used to investigate the effect of each adolescent anthropometric indices in cIMT in early adulthood. Accordingly, three adjusted models were constructed: Model 1 was adjusted for age, family history of CVD, and smoking; Model 2: further adjusted for systolic blood pressure, total cholesterol, and fasting plasma glucose; Model 3: Model 2 + adulthood relevant anthropometric measurements (i.e., for waist circumference, adulthood waist circumference was included as another covariate). The area under the receiver-operating characteristic curve (AUC) and 95% confidence interval was used to evaluate the predictive ability of each adolescent anthropometric indices, followed by a comparison of all adolescent anthropometric indices using the Delong test (19). In addition, Youden’s index (sensitivity + specificity-1) was used to obtain the best cut-off from the ROC curve. The values for the maximum of the Youden’s index were considered the optimal cut-off points (20). All statistical analyses were conducted using SPSS version 20 (SPSS, Chicago, IL, USA) regarding a two-tailed *P*-value of < 0.05 as a significant.

## Results

At the recruitment time, the study population included 454 boys and 421 girls with mean ages of 13.3, and 13.5, years old respectively; the corresponding values for BMI were 19.6 and 20.1 (Kg/m^2^), respectively. The characteristics and cardiometabolic profile of the participants according to gender are shown in Table 1. At baseline, girls had significantly higher WC, HC, WHtR, TC, TG, and LDL-C than boys. However, boys were taller compared to girls and had higher values of WHR, SBP, and HLD-C. No significant differences were seen regarding age, weight, BMI, DBP, and FPG between boys and girls.

**TABLE 1:** Participants characteristics at baseline and the end of follow-up.

Variable	Male (*n* = 454)	Female (*n* = 421)	*p*-value
**Baseline**			
Age (years)	13.3 ± 2.1	13.5 ± 2.2	0.13
Weight (kg)	49.8 ± 16.2	48.4 ± 11.9	0.14
Height (cm)	157.5 ± 14.4	154.5 ± 8.7	<0.001
BMI (kg/m^2^)	19.6 ± 4.1	20.1 ± 3.9	0.09
WC (cm)	67.5 ± 11.1	69.2 ± 9.1	0.02
HC (cm)	82 ± 11.3	88.8 ± 10.5	<0.001
WHR	0.82 ± 0.06	0.78 ± 0.06	<0.001
WHtR	0.43 ± 0.05	0.44 ± 0.05	<0.001
SBP (mmHg)	106.1 ± 11.7	102.8 ± 11.2	<0.001
DBP (mmHg)	70.1 ± 9.4	70.5 ± 9.1	0.57
FPG (mg/dl)	89.1 ± 7.5	88.1 ± 7.9	0.08
Total cholesterol (mg/dl)	166.1 ± 34.3	171.4 ± 30.8	0.02
Triglycerides (mg/dl)	91.5 (67–127.7)	101 (77–139)	<0.01
HDL-C (mg/dl)	44 ± 10.6	42.4 ± 10.4	0.02
LDL-C (mg/dl)	100.3 ± 30.1	105.8 ± 27.7	<0.01
**End of follow-up**			
Age of cIMT measured (years)	31.9 ± 2.3	32.4 ± 2.4	<0.01
Weight (kg)	84.9 ± 15.9	66 ± 12.3	<0.001
Height (cm)	176.4 ± 6.6	160.4 ± 5.7	<0.001
BMI (kg/m^2^)	27.2 ± 4.6	25.7 ± 4.8	<0.001
WC (cm)	94.4 ± 11.6	84.8 ± 10.6	<0.001
HC (cm)	99.8 ± 8.1	100.9 ± 8.9	0.06
WHR	0.94 ± 0.05	0.84 ± 0.07	<0.001
WHtR	0.53 ± 0.06	0.52 ± 0.06	0.16
cIMT (mm)	0.54 ± 0.10	0.57 ± 0.09	<0.001
Smoking no. (%)	157 (34.6)	25 (5.9)	<0.001
Family history CVD no. (%)	12 (2.6)	11 (2.6)	0.98

Data are given as the mean (SD) or median (IQ 25–75) unless otherwise indicated. BMI, body mass index; WC, waist circumference; HC, hip circumference; WHR, waist-to-hip ratio; WHtR, waist-to-height ratio; SBP, systolic blood pressure; DBP, diastolic blood pressure; FPG, fasting plasma glucose; HDL-C, high-density lipoprotein cholesterol; LDL-C, low-density lipoprotein cholesterol; CVD, cardiovascular disease; cIMT, carotid intima-media thickness.

After the median (IQR) follow-up duration 18.2 (17.8–18.8) years, cIMT was measured in early adulthood (the mean age was 32.1 ± 2.3 years), and the mean value was 0.55 ± 0.09 mm. As shown in Table 1, at the end of the follow-up, the mean age of cIMT measurement was significantly higher among females than males. Also, females had significantly higher mean values of cIMT than males, however, the values of weight, BMI, WC, and WHR were higher in the latter. The males were more likely to be smoker than females. Although, no significant differences in WHtR and prevalence of family history of CVDs between genders were found.

The characteristics of respondents and non-respondents are provided in Supplementary Table 1. The respondents were younger, constituted a greater proportion of males, and had lower values of WC, HC, and DBP, however, no significant differences were found for other baseline characteristics between them.

Table 2 demonstrates the odds ratio and 95% confidence intervals of each adolescent anthropometric indices for high cIMT (> 90th percentile) in early adulthood using multivariable logistic regression. In females, no significant association were found between each adolescent anthropometric indices and risk of high cIMT in early adulthood even in model 1. In male, all adolescent anthropometric indices were significantly associated with high cIMT in early adulthood in model 1 with ORs ranging from 1.35 for WHR to 1.60 for WC; after controlling multiple cardiometabolic risk factors (Model 2), all adolescent anthropometric indices were significantly associated with high cIMT in early adulthood, with ORs ranging from 1.33 for WHR to 1.63 for WC (all *p*-values < 0.05 for both models). After considering adulthood relevant anthropometric measurements as a confounder (model 3), all of the associations significantly attenuated excluding WC [1.48 (95% CI: 1.04–2.10)] and WHR [1.28 (95% CI: 0.99–1.66), *p*-value = 0.06]. According to age- and sex-stratified analysis, no significant association was found between each anthropometric indices and risk of high cIMT in early adulthood except in boys aged 15–17 years old. A 1-SD increase in each adolescent anthropometric indices in boys aged 10–14 years old, were significantly increased the risk of high cIMT in model 3, the corresponding values for BMI, WC, WHR, and WHtR were 1.81 (1.13–2.92), 2.04 (1.31–3.18), 1.36 (1.02–1.83), and 1.85 (1.22–2.80), respectively.

**TABLE 2:** Sex and age group stratified association of adolescent anthropometric indices with high carotid intima-media thickness (≥ 90th percentiles) in early adulthood.

	Male		Female	
	Odds ratio (95% CI)	*P*-value	Odds ratio (95% CI)	*P*-value
**Total**				
**Body mass index**				
Model 1	1.43 (1.09–1.87)	<0.01	1.21 (0.90–1.64)	0.20
Model 2	1.43 (1.05–1.94)	0.02	1.25 (0.90–1.72)	0.18
Model 3	1.22 (0.83–1.79)	0.31	0.84 (0.55–1.28)	0.42
**Waist circumference**				
Model 1	1.60 (1.24–2.07)	<0.001	1.20 (0.87–1.65)	0.27
Model 2	1.63 (1.22–2.19)	<0.01	1.19 (0.85–1.68)	0.31
Model 3	1.48 (1.04–2.10)	0.03	0.95 (0.65–1.39)	0.78
**Waist-to-hip ratio**				
Model 1	1.35 (1.05–1.74)	0.02	1.20 (0.89–1.61)	0.22
Model 2	1.33 (1.03–1.71)	0.03	1.17 (0.86–1.58)	0.32
Model 3	1.28 (0.99–1.66)	0.06	1.15 (0.84–1.56)	0.38
**Waist-to-height ratio**				
Model 1	1.42 (1.11–1.82)	<0.01	1.18 (0.89–1.55)	0.25
Model 2	1.41 (1.07–1.87)	0.01	1.18 (0.88–1.59)	0.26
Model 3	1.33 (0.95–1.85)	0.10	0.97 (0.69–1.35)	0.85
**10–14 years old**				
**Body mass index**				
Model 1	1.81 (1.27–2.59)	<0.01	1.16 (0.78–1.75)	0.46
Model 2	1.89 (1.25–2.84)	<0.01	1.21 (0.79–1.86)	0.39
Model 3	1.81 (1.13–2.92)	0.01	0.69 (0.38–1.25)	0.21
**Waist circumference**				
Model 1	1.95 (1.38–2.76)	<0.001	1.20 (0.80–1.80)	0.38
Model 2	2.00 (1.36–2.95)	<0.001	1.18 (0.76–1.83)	0.45
Model 3	2.04 (1.31–3.18)	<0.01	0.89 (0.54–1.46)	0.65
**Waist-to-hip ratio**				
Model 1	1.39 (1.05–1.86)	0.02	1.24 (0.87–1.77)	0.23
Model 2	1.37 (1.03–1.84)	0.03	1.17 (0.81–1.70)	0.39
Model 3	1.36 (1.02–1.83)	0.04	1.16 (0.80–1.68)	0.44
**Waist-to-height ratio**				
Model 1	1.76 (1.27–2.46)	<0.01	1.16 (0.82–1.63)	0.41
Model 2	1.76 (1.22–2.54)	<0.01	1.17 (0.80–1.69)	0.42
Model 3	1.85 (1.22–2.80)	<0.01	0.88 (0.57–1.36)	0.58
**15–17 years old**				
**Body mass index**				
Model 1	1.05 (0.68–1.62)	0.83	1.28 (0.82–2.00)	0.29
Model 2	1.06 (0.64–1.76)	0.81	1.29 (0.79–2.12)	0.30
Model 3	0.73 (0.36–1.49)	0.38	1.03 (0.54–1.95)	0.93
**Waist circumference**				
Model 1	1.21 (0.80–1.82)	0.37	1.21 (0.72–2.04)	0.48
Model 2	1.27 (0.79–2.05)	0.32	1.21 (0.68–0.2.13)	0.52
Model 3	0.91 (0.49–1.69)	0.77	1.03 (0.54–1.97)	0.93
**Waist-to-hip ratio**				
Model 1	1.36 (0.79–2.33)	0.26	1.14 (0.65–2.02)	0.65
Model 2	1.47 (0.81–2.67)	0.20	1.13 (0.63–2.02)	0.68
Model 3	1.24 (0.63–2.43)	0.54	1.14 (0.63–2.06)	0.67
**Waist-to-height ratio**				
Model 1	1.07 (0.72–1.60)	0.73	1.21 (0.75–1.93)	0.44
Model 2	1.10 (0.69–1.73)	0.70	1.20 (0.72–2.01)	0.47
Model 3	0.83 (0.46–1.50)	0.54	1.07 (0.60–1.90)	0.81

Odds ratio expressed per 1-SD increment. Model 1: adjusted for age and adulthood family history CVD and smoking status. Model 2: model 1 + systolic blood pressure, total cholesterol, and fasting blood sugar. Model 3: model 2 + adulthood relevant anthropometric measurement (i.e., for waist circumference, adulthood waist circumference was included in adjustment model).

*P*-value < 0.05 is significant.

The early adolescent males were generally had better cardiometabolic health status both at the baseline and at the end of the follow up compared to late adolescent ones, however, no differences were found in term of cIMT between these groups (Supplementary Table 2). Among male participants, in the early adolescent group, regardless of baseline anthropometric measures, no significant associations were found for other confounder/mediators, however for late adolescent group the adulthood cholesterol level was significantly associated with high cIMT. Moreover, among female, in the early adolescent group, baseline FPG and the adulthood general and central adiposity measures were remained as significant predictors of high cIMT, however, no significant association were found for confounder/mediators in later adolescent ones (data not shown).

Sex and age group stratified association of adolescent anthropometric indices with cIMT (in mm) in early adulthood is also shown in Supplementary Table 3 using linear regression analysis. Accordingly, the result among males were generally in the line with the main analysis conducted using multivariate logistic regression. Importantly among females, however, in contrast to logistic regression analysis we found a significant association between WC, WHR, and WHtR and higher value of high cIMT in model 1, the relationship that remained significant only for WHtR after further adjustment for cardiometabolic risk factors (model 2).

**FIGURE 1:**
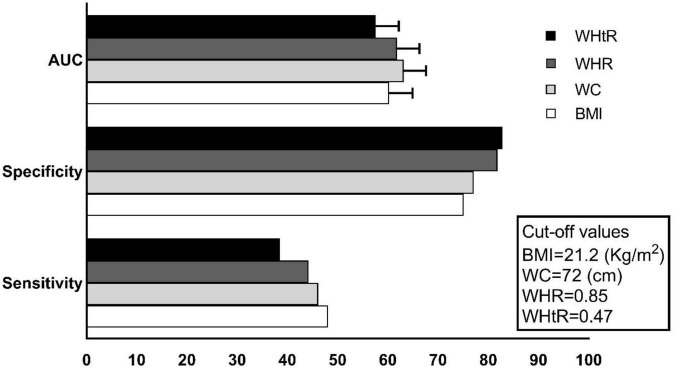
The area under the curves (AUCs*100), sensitivity (%), specificity (%), and cut-off values of adolescent anthropometric indices for predicting high cIMT in boys.

Figure 1 illustrates the area under the receiver operating curve (AUC) and 95% confidence intervals, sensitivity, specificity, and cut-off values of each adolescent anthropometric indices in boys for predicting high cIMT in early adulthood. The AUC levels ranged from 0.632 (95% CI: 0.585–0.676) for WC to 0.576 (95% CI: 0.529–0.622) for WHtR, the corresponding cut-off points were 72 (cm), and 0.47, respectively. Supplementary Table 4 shows the comparison of AUCs of adolescent anthropometric indices in boys. No significant differences were observed between the AUCs of adolescent anthropometric indices (all *p*-values > 0.05). However, we found a signal for higher discriminatory power of WC compared to BMI and WHtR in boys (*p*-value = 0.07).

## Discussion

This community-based study of Iranian adolescents with a two-decade follow-up period showed that anthropometric indices, including BMI, WC, WHR, and WHtR, can predict the risk of high cIMT in early adulthood among boys; however, these associations were not found in girls. Considering adjustment for a large set of covariates (including obesity mediators, and corresponding adulthood anthropometric measurements) among boys WC and WHR were significant predictors, although these relationships were tended to be significant for the latter. Importantly, among early but not late adolescent males, all of the anthropometric measurements were significantly associated with high cIMT. Among Iranian females, we found generally in linear regression analysis, a significant association between the higher value of WC and WHtR with cIMT measurement in adulthood, however, these risks reach to null after considering adult anthropometric measures. Regarding the discriminatory power of anthropometric measurement as assessed by AUC, no differences were found between the predictive performance of anthropometric measures in boys for high cIMT, however, a signal for higher discriminatory power of WC compared with BMI and WHtR were found (*p*-value = 0.07).

A number of prospective studies have demonstrated a link between pediatric obesity by measuring BMI, which represents general obesity, or triceps skinfold thickness (TSF), and the risk of thicker cIMT in adulthood (Table 3). In accordance with our findings, the international Childhood Cardiovascular Cohort (i3C) Consortium has shown that childhood BMI significantly predicted thicker cIMT in adulthood (21). Furthermore, the pooled analysis of four longitudinal studies, including the Bogalusa Heart Study, the Insulin Study, Childhood Determinants of Adult Health, and the Cardiovascular Risk in Young Finns Study, have demonstrated that childhood BMI was associated with high cIMT (> 90th percentile) in adulthood after two decades of follow up (22).

**TABLE 3:** Description of papers that evaluated the association between childhood adiposity and carotid intima-media thickness (cIMT) in adulthood.

References	Study cohort, country	Participants (*n*)	Age at baseline (year)	Adiposity index	Follow-up duration (year)	Age at follow-up (years)	Adulthood cIMT (mm)	Main finding(s)	Adjustment variables
Davis et al. (34)	Muscatine Heart Study, United States	725 (346 males)	8–11	BMI TSF	–	33–42	0.79 ± 0.12 for men 0.72 ± 0.10 for women	1-SD increase in childhood BMI was associated with 47% increases risk of high cIMT (> 75th percentile) in women	Childhood age
Oren et al. (13)	Atherosclerosis Risk in Young Adults (ARYA), Netherlands	750 (352 males)	12–16	BMI	–	27–30	0.49 ± 0.05 for men 0.48 ± 0.05 for women	1-SD increase in adolescent BMI was associated with 2.3 μm increase in cIMT in young adults, but this association became non-significant when adult BMI adjusted	cIMT reader, childhood gender, lumen diameter, age, BP, pubertal stage, and adult BP, lipid levels, and BMI
Raitakari et al. (32)	Cardiovascular Risk in Young Finns Study, Finland	2,229 (1,005 males)	3–18	BMI	21	24–39	0.64 ± 0.11 for men 0.61 ± 0.09 for women	Greater BMI in childhood was associated with thicker cIMT in men (β = 0.013) and women (β = 0.04). In sub-group analysis, the association was only seen in the 12–18 years old These associations became non-significant when adult BMI adjusted	Childhood and adulthood age, gender, LDL-C, SBP, and smoking
Li et al. (35)	Bogalusa Heart Study, United States	486 (191 males)	4–17	BMI	22.2	25–37	0.757 ± 0.073 for men 0.719 ± 0.091 for women	1-SD increase in childhood BMI was associated with 67% increases risk of high cIMT (highest quartile of cIMT *z*-score)	
Freedman et al. (36)	Bogalusa Heart Study, United States	513 (203 males)	Six measure-ments between 4–35 years	BMI TSF		23–40	0.736 in total	BMI and TSF were significantly associated with high cIMT (≥ 90th percentile) in adulthood. Associations were stronger in women than men. This association attenuated when adult BMI was adjusted. The association was also significant among overweight children who remained obese in adulthood.	Race, age, gender, adult adiposity indices
Ferreira et al. (37)	Amsterdam Growth and Health Longitudinal Study (AGAHLS), Netherlands	159 (75 males)	13–16	BMI TSF DXA	24	36	0.62 ± 0.010 for men 0.63 ± 0.10 for women	Total body fatness and truncal subcutaneous fat accumulation (the latter in boys only) during childhood were positively associated with cIMT in adulthood	Gender, TC/HDL-C ratio, TG, HbA1C, resting heart rate and cardiopulmonary fitness in childhood, height and pulse pressure in adulthood
Juonala et al. (24)	Cardiovascular Risk in Young Finns Study, Finland	2,260	3–18	BMI		29–34	0.622 in total	BMI in childhood correlated with adult cIMT, this association became non-significant when adjusted for adult BMI. The association was significant among those who gained weight and among overweight children who remained obese in adulthood. BMI measured at 12, 15, and 18 years old was only associated with cIMT in adulthood	Adult BMI
Li et al. (38)	Bogalusa Heart Study, United States	868 (365 males)	4–17	BMI	26.4	25–44	0.85 ± 0.016 for men 0.77 ± 0.13 for women	BMI only in black women was significant predictor of cIMT in young adults	Gender, race, SBP, HDL-C, LDL-C, TG
Freedman et al. (23)	Bogalusa Heart Study, United States	1,142 (492 males)	7–13	BMI		36	0.87 ± 0.20 for men 0.77 ± 0.14 for women	Childhood BMI levels were related to cIMT in adulthood. This association attenuated when adult BMI was adjusted.	Gender, age, race, adult BMI
Juonala et al. (21)	International Childhood Cardiovascular Cohort (i3C) Consortium	4,380 (2,002 males)	3–18	BMI	22.4	20–45		Childhood BMI at or after 9 years of age was associated with higher cIMT in adulthood	
Juonala et al. (39)	Cardiovascular Risk in Young Finns Study, Finland	1,809 (794 males)	3–18	BMI	27	24–39	–	Obesity was associated with 6-year change in adulthood cIMT. This association remained significant when adjusted for adulthood risk score and genotype score.	Childhood and adulthood HDL-C, physical activity, fruit consumption, and genotype score
Juonala et al. (18)	International Childhood Cardiovascular Cohort (i3C) Consortium	6,328 (2,961 males)	3–18	BMI	23.1	23–46	0.65 ± 0.12 in total	Overweight children who remained obese in adulthood had an increased risk of high cIMT (≥ 90th percentile). In sub-group analysis, the association was seen only in boys. The risks of high cIMT among overweight or obese children who became non-obese by adulthood were similar to those among persons who were never obese.	Gender, age, height, follow-up length, and cohort.
Magnussen et al. (40)	Bogalusa Heart Study and the Cardiovascular Risk in Young Finns Study	1,781 (784 males)	9–18	BMI	14–27	24–41		BMI alone was as good as and in some cases superior to dichotomous pediatric MetS definitions in predicting high cIMT in adulthood	Length of follow-up, cohort, and all other MetS components
Huynh et al. (41)	Childhood Determinants of Adult Health (CDAH), Australia	2,328 (1,150 males)	7–15	BMI TSF	20	26–36	0.58 ± 0.089 in total	Childhood body size or adiposity was associated with adulthood cIMT	Childhood and adulthood weight, height, childhood BP and age of menarche
Su et al. (42)	Young Taiwanese Cohort (YOTA) Study, Taiwan	789 (313 males)	6–18	BMI	8.5	21.32	0.438 ± 0.048 in total	Overweight/obese children and adolescents who remain obese or overweight in adulthood had higher risk of high cIMT (> 95th percentile) in adulthood	Gender, age, fasting glucose, TC, smoking, alcohol habit, household income
Koskinen et al. (43)	Cardiovascular Risk in Young Finns Study, Finland	1,617 (766 males)	9–24	BMI TSF	21–25	30–45		Children who were either overweight or metabolically abnormal had similar increased risk of elevated cIMT in adulthood	FBS, TG, HDL-C, LDL-C cholesterol, SBP in childhood, and BMI in adulthood as well as family history of coronary artery disease
Johnson et al. (44)	MRC National Survey of Health and Development Study, United Kingdom	1,273 (604 males)	2–20	BMI Height		60–64	0.667 mm	Higher BMI associated with thicker cIMT in men only, and only at exposure ages 4 and 20	Fathers’ education levels, BMI in childhood and adulthood, SBP, pulse pressure, HbA1C in adulthood as well as total energy intake in childhood
Ceponiene et al. (27)	Kaunas Cardiovascular Risk Cohort study, Lithuania	380 (168 males)	12–13	BMI	35	48–49	0.66 ± 0.11 for men 0.61 ± 0.08 for women	Higher BMI in childhood is associated with thicker cIMT in adulthood only in women. This association became non-significant when adult mediators adjusted	BMI, SBP, and sexual maturity score in childhood as well as BMI, SBP, HDL-C, LDL-C, smoking, and educational levels in adulthood
Yan et al. (25)	Beijing blood pressure cohort, China	1,252 (692 males)	6–18	BMI	22.9	27–42	0.54 ± 0.05 for men 0.5 ± 0.04 for women	Childhood BMI predicted high cIMT (> 75th percentile) in adulthood, this association became non-significant when adult BMI and SBP were adjusted. In addition, incremental BMI from childhood to adulthood predicted high cIMT in adulthood.	Follow-up length, TG, HDL-C, LDL-C, smoking, drinking, physical inactivity, and family history of stroke and coronary heart disease in adulthood
Du et al. (45)	Bogalusa Heart Study, United States	1,052 (455 males)	4–19	BMI	26.5	19–52	0.87 ± 0.19 for men 0.77 ± 0.13 for women	The association between childhood obesity and cIMT was only seen in individuals with low adiponectin levels.	Gender, race, age, LDL-C, HDL-C, TG, SBP in childhood as well as smoking in adulthood
Hao et al. (46)	Georgia Stress and Heart study, United States	626	5–18	BMI		24		Childhood trajectory of BMI was associated with cIMT in adulthood even after adjustment for adulthood BMI, but the associations for IMT were not significant after adjustment for BMI at baseline	Gender, race, age, father’s education level, SBP and DBP, BMI in adulthood, BMI in childhood
Koskinen et al. (22)	International Childhood Cardiovascular Cohort (i3C) Consortium	2,893 (1,331 males)	12–18	BMI	23.4			Childhood BMI associated with high cIMT (> 90th percentile) even after lipid biomarkers adjustments	Gender, age, blood pressure, smoking, LDL-c, HDL-c, TG
Buscot et al. (26)	Cardiovascular Risk in Young Finns Study, Finland	2,631 (1,208 males)	6–18	BMI	30	34–49		Childhood adiposity was associated with high cIMT (> 90th percentile). The effect of youth obesity on the risk of high cIMT may not be reversible even with the normalization of high BMI in later life	Gender, year of birth, family history of CVD, socio-economic status, and physical activity level in adulthood
Hosseinpanah et al. (12)	Tehran lipid and glucose study (TLGS), Iran	1,295 (670 males)	3–18	BMI	18	29.7 ± 4	0.55 ± 0.1	Childhood BMI associated with thicker cIMT in adulthood	Gender, age, family history of CVD, smoking, adulthood BMI
Tasdighi et al. (6)	Tehran lipid and glucose study (TLGS), Iran	1,220 (631 males)	10.9 ± 4	BMI	18	30 ± 3.8	0.55 ± 0.1	Children with metabolically unhealthy obese phenotype was the only ones that had an increased risk of high CIMT incidence in early adulthood	Gender, age, family history CVD, smoking, adulthood BMI, and LDL

BMI, body mass index; TSF, triceps skinfold; DXA, dual energy X-ray absorptiometry; BP, blood pressure; SBP, systolic blood pressure; TC, total cholesterol; TG, triglyceride; HDL-C, high-density lipoprotein cholesterol; LDL-C, low-density lipoprotein cholesterol; HbA1C, hemoglobin A1C; MetS, metabolic syndrome; FBS, fasting blood pressure; CVD, cardiovascular disease.

The result of our study has shown that the predictive power of all anthropometric indices for the prediction of high cIMT in early adulthood was attenuated and only remained significant in WC among males after adjustment of adulthood relevant anthropometric measurements. Summarizing the results of a majority of longitudinal cohort studies, the positive associations between childhood BMI and thicker cIMT in adulthood were generally reduced after adjustment for adulthood BMI (23) or became non-significant (13, 24, 25). These findings may provide some clues that the patterns of adiposity anthropometric indices trajectories from childhood to adulthood also plays an important role in predicting the thicker cIMT in adulthood along with childhood adiposity. Juonala et al. have demonstrated that childhood adiposity was associated with thicker cIMT in adulthood, but this association can be attributed to significant changes in BMI from childhood to adulthood (24). They also reported that the corresponding adulthood cIMT values in individuals who were obesity gainers from childhood to adulthood compared to individuals who had been persistently obese were comparable. As well, the cIMT values tended to be lower, yet comparable, in individuals who had been consistently non-obese and those who had been obesity reducers from childhood to adulthood. However, The Cardiovascular Risk in Young Finns Study has indicated that the effect of childhood obesity on the risk of high cIMT, even with the normalization of high BMI in later life, has not been completely eliminated (26). To our knowledge, no study compare the association between central adiposity measures with high cIMT in early adulthood.

We also found that the association between adolescent anthropometric indices and risk of high cIMT in early adulthood was observed only among boys. In line with our findings, in an I3C consortium of 6,380 participants, Juonala et al. have demonstrated that the mentioned association was seen in boys (18). In contrast, another I3C Consortium has shown that no significant differences between gender were observed for the predictive power of childhood BMI for the risk of high cIMT in adulthood (21). Additionally, Ceponiene et al. have indicated that higher childhood BMI was associated with thicker cIMT in women adults; that this association was not independent of adult cardiometabolic risk factors (27). In the current study when we examined cIMT as a continuous rather than categorical variable as the outcome we found that Tehranian girls with higher WC had significantly higher values of cIMT in the presence of cardiometabolic risk factors. Considering the observational nature of our study, it is not possible for us to explain the sex difference in the association between anthropometric measures and subclinical atherosclerosis in early adulthood; however, previously we reported the prominent role of general and central adiposity measures in the development of CVD among adult men rather than women (28).

Studies that have evaluated childhood obesity and the risk of high cIMT in adulthood have mainly measured BMI as an obesity indicator (as shown in Table 3), which may fail to reflect central obesity (29). The results from NHANES III, including the 15,184 participants aged 18–90 years old, showed that central adiposity increased the risk of CV mortality among individuals with normal BMI (30). Wang et al. in the cohort of Chinese children, demonstrated that compared with persistently normal WC, gaining in abdominal obesity, whether as incident or persistent, were significantly associated with the short-term risk of high cIMT during a 2-year follow-up in childhood (31).

As an important finding, in our subgroup analysis by age group, we found that the unfavorable impact of increasing value for general and central adiposity indices on cIMT thickening were more prominent among early adolescent boys. According to the findings of the I3C consortium, Jounala et al. have shown that childhood BMI after age 9 years was significantly associated with high cIMT in adulthood (21). In addition, Raitakari et al. have demonstrated that greater BMI in adolescents aged 12–18 years old was significantly associated with thicker cIMT in adulthood (32). Importantly, other cardiometabolic risk factors besides anthropometric measurements specially baseline fasting glucose among early adolescent girl and adulthood cholesterol level among late adolescent boys had significant association with high cIMT in our study (data not shown); the role of these obesity mediators in the development of atherosclerosis were addressed in other studies as well (21, 33).

In light of these findings, it is reasonable to conclude that childhood adiposity beginning in early puberty, may cause progressive damage to the common carotid artery in adulthood.

### Strengths and limitations

Our study has several strengths. It is important to point out that not only childhood cardiometabolic confounders but also adulthood relevant anthropometric indices were considered in our analysis. However, there are also some limitations to be considered. First, although we adjusted major confounding variables in our analyses, residual or unmeasured factors, such as physical activity, dietary intakes, adipokines and cytokines, pubertal status, and genetic background were not considered in our data analysis. Second, we should point out that the respondents were generally healthier than non-respondents, however, the magnitude of the difference between these groups were not clinically important. Third, we conducted this study among Tehranian adolescents, so we cannot generalize our findings to other parts of the country, especially the rural zones.

## Conclusion

The present study was the first to evaluate the predictive power of general and central childhood adiposity indices and the risk of high cIMT in early adulthood not only in the Eastern Mediterranean Region but also worldwide. Childhood general and central anthropometric indices significantly predicted the high cIMT as the surrogate marker of CVD in early adulthood only among male adolescents without any differences in predictive power between the anthropometric indices. Following further adjustment for adulthood relevant anthropometric indices, generally, all of the male anthropometric indices except WC and WHR were no longer significant predictors of high cIMT in early adulthood. No superiority in the discriminatory ability for high cIMT among anthropometric indices was found in adolescent males. The predictive ability of high cIMT in early adulthood were prominent among pre-pubertal boys.

## Data availability statement

The raw data supporting the conclusions of this article will be made available by the authors, without undue reservation.

## Ethics statement

The studies involving human participants were reviewed and approved by the Ethics Committee of the Research Institute for Endocrine Sciences (RIES) of Shahid Beheshti University of Medical Sciences. Written informed consent to participate in this study was provided by the participants’ legal guardian/next of kin.

## Author contributions

FHa contributed to the conception and design of the study. PD contributed to the acquisition of the ultrasound data. MM analyzed the data. GA and AN took lead in the literature review and writing the manuscript in consultation with FHa. All authors reviewed and approved the final draft of the manuscript.
