# Supplementary material for: Diagnostic performance of different anthropometric indices among Iranian adolescents for intima media thickness in early adulthood: A prospective study and literature review
Source: Front Nutr. 2023 Feb 28;10:1098010. doi: 10.3389/fnut.2023.1098010 (PMC10012864; doi:10.3389/fnut.2023.1098010)
Supplement: Supplementary file 1 [file Data_Sheet_1.docx]

| **Supplementary table 1.** Characteristics of study respondents and non-respondent in the Tehran lipid and glucose study | | | | |
| --- | --- | --- | --- | --- |
| Variable | Respondent (n=875) | Non respondent (n=1768) | P-value |  |
| Age (year) | 13.4 ± 2.1 | 13.7 ± 2.8 | 0.02 |  |
| Male (%) | 448 (51.7) | 825 (46.7) | 0.01 |  |
| BMI (kg/m^2^) | 19.8 ± 4 | 20 ±4.3 | 0.20 |  |
| WC (cm) | 68.3 ± 10.2 | 69.2 ± 10.8 | 0.04 |  |
| HC (cm) | 85.3 ± 11.4 | 86.6 ± 11.4 | <0.01 |  |
| WHR | 0.80 ± 0.07 | 0.80 ± 0.07 | 0.30 |  |
| WHtR | 0.44 ± 0.06 | 0.44 ± 0.06 | 0.22 |  |
| SBP (mmHg) | 104.5 ± 11.6 | 105.1 ± 11.7 | 0.20 |  |
| DBP (mmHg) | 70.4 ± 9.3 | 71.1 ± 9.2 | 0.04 |  |
| FPG (mg/dl) | 88.7 ± 7.7 | 88.8 ± 11.4 | 0.66 |  |
| Total Cholesterol (mg/dl) | 168.9 ± 32.8 | 167.7 ± 32.4 | 0.38 |  |
| Triglycerides (mg/dl) | 92 (72 - 135) | 96 (71 - 133) | 0.63 |  |
| HDL-C (mg/dl) | 43.2 ± 10.6 | 43.1 ± 10.3 | 0.78 |  |
| LDL-C (mg/dl) | 103.2 ± 29.2 | 102.5 ± 28.3 | 0.59 |  |
| Data are given as the mean (SD) or median (IQ 25-75) unless otherwise indicated.  Abbreviations: BMI, body mass index; WC, waist circumference; HC, hip circumference; WHR, waist-to-hip ratio; WHtR, waist-to-height ratio; SBP, systolic blood pressure; DBP, diastolic blood pressure; FPG, fasting plasma glucose; HDL-C, high-density lipoprotein cholesterol; LDL-C, low-density lipoprotein cholesterol | | | | |

| **Supplementary table 2.** Boys characteristics stratified by age group at baseline and at the end of follow-up | | | |  |
| --- | --- | --- | --- | --- |
|  | 10-14 years old (n=305) | 15-17 years old (n=149) | P-value | |
| **Baseline** |  |  |  | |
| Age (year) | 12.1 ± 1.4 | 15.8 ± 0.7 | <0.001 | |
| Weight (kg) | 43.4 ± 13.1 | 63 ± 13.8 | <0.001 | |
| Height (cm) | 150.8 ± 12.2 | 171.2 ± 6.9 | <0.001 | |
| BMI (kg/m^2^) | 18.7 ± 3.7 | 21.4 ± 4.3 | <0.001 | |
| WC (cm) | 64.7 ± 10 | 73.4 ± 11 | <0.001 | |
| HC (cm) | 78 ± 10.1 | 90.3 ± 8.8 | <0.001 | |
| WHR | 0.83 ± 0.07 | 0.81 ± 0.06 | <0.01 | |
| WHtR | 0.43 ± 0.05 | 0.43 ± 0.06 | 0.91 | |
| SBP (mmHg) | 104 ± 11.3 | 110.3 ± 11.6 | <0.001 | |
| DBP (mmHg) | 69 ± 9.2 | 72.4 ± 9.4 | <0.001 | |
| FPG (mg/dl) | 89.3 ± 7.5 | 88.6 ± 7.3 | 0.32 | |
| Total Cholesterol (mg/dl) | 168.4 ± 32.2 | 161.4 ± 37.9 | 0.04 | |
| Triglycerides (mg/dl) | 88 (65 – 118) | 97 (72.5 – 139) | 0.01 | |
| HDL-C (mg/dl) | 46 ± 11 | 39.9 ± 8.5 | <0.001 | |
| LDL-C (mg/dl) | 101.5 ± 28.5 | 97.9 ± 33.4 | 0.23 | |
| **End of follow up** |  |  |  | |
| Age of cIMT measured (years) | 30.8 ± 1.8 | 34.3 ± 1.4 | <0.001 | |
| Weight (kg) | 84.6 ± 16 | 85.6 ± 15.8 | 0.54 | |
| Height (cm) | 176.4 ± 6.8 | 176.4 ± 6.1 | 0.93 | |
| BMI (kg/m^2^) | 27.2 ± 4.7 | 27.4 ± 4.5 | 0.58 | |
| WC (cm) | 93.8 ± 11.5 | 95.6 ± 11.7 | 0.11 | |
| HC (cm) | 99.6 ± 8.1 | 100.2 ± 8.3 | 0.49 | |
| WHR | 0.94 ± 0.06 | 0.95 ± 0.05 | 0.02 | |
| WHtR | 0.53 ± 0.06 | 0.54 ± 0.06 | 0.13 | |
| SBP (mmHg) | 113.1 ± 10.9 | 112.8 ± 11.8 | 0.79 | |
| DBP (mmHg) | 76.2 ± 8.7 | 77.9 ± 8.9 | 0.06 | |
| FPG (mg/dl) | 90.6 ± 11.8 | 92.2 ± 12.4 | 0.18 | |
| Total Cholesterol (mg/dl) | 179.2 ± 36.1 | 187.3 ± 40.4 | 0.03 | |
| Triglycerides (mg/dl) | 116 (85 – 171) | 142 (98 – 195) | 0.01 | |
| HDL-C (mg/dl) | 43.6 ± 10.1 | 41.7 ± 8.4 | 0.05 | |
| LDL-C (mg/dl) | 108 ± 29.9 | 114 ± 36.2 | 0.07 | |
| cIMT (mm) | 0.53 ± 0.09 | 0.55 ± 0.01 | 0.13 | |
| Data are given as the mean (SD) or median (IQ 25-75) unless otherwise indicated.  Abbreviations: BMI, body mass index; WC, waist circumference; HC, hip circumference; WHR, waist-to-hip ratio; WHtR, waist-to-height ratio; SBP, systolic blood pressure; DBP, diastolic blood pressure; FPG, fasting plasma glucose; HDL-C, high-density lipoprotein cholesterol; LDL-C, low-density lipoprotein cholesterol | | | |  |

**Supplementary table 3.** Sex and age group stratified association of adolescent anthropometric indices with carotid intima-media thickness in early adulthood

|  | **Male** | | **Female** | |
| --- | --- | --- | --- | --- |
|  | Beta coefficient (SE) | P-value | Beta coefficient (SE) | P-value |
| **Total** |  |  |  |  |
| Body mass index (kg/m^2^) |  |  |  |  |
| Model 1 | 0.021 (0.005) | <0.001 | 0.009 (0.005) | 0.09 |
| Model 2 | 0.018 (0.005) | <0.01 | 0.009 (0.006) | 0.12 |
| Model 3 | 0.013 (0.007) | 0.04 | -0.007 (0.007) | 0.28 |
| Waist circumference (cm) |  |  |  |  |
| Model 1 | 0.025 (0.005) | <0.001 | 0.012 (0.006) | 0.03 |
| Model 2 | 0.023 (0.005) | <0.001 | 0.010 (0.006) | 0.07 |
| Model 3 | 0.020 (0.006) | <0.01 | 0.001 (0.006) | 0.89 |
| Waist-to-hip ratio |  |  |  |  |
| Model 1 | 0.019 (0.005) | <0.001 | 0.010 (0.005) | 0.04 |
| Model 2 | 0.016 (0.005) | <0.01 | 0.008 (0.005) | 0.10 |
| Model 3 | 0.014 (0.005) | <0.01 | 0.007 (0.005) | 0.18 |
| Waist-to-height ratio |  |  |  |  |
| Model 1 | 0.020 (0.005) | <0.001 | 0.011 (0.005) | 0.03 |
| Model 2 | 0.017 (0.005) | <0.01 | 0.010 (0.005) | 0.04 |
| Model 3 | 0.016 (0.006) | <0.01 | 0.002 (0.005) | 0.75 |
| **10-14 years old** |  |  |  |  |
| Body mass index (kg/m^2^) |  |  |  |  |
| Model 1 | 0.027 (0.006) | <0.001 | 0.009 (0.007) | 0.18 |
| Model 2 | 0.024 (0.006) | <0.001 | 0.008 (0.007) | 0.27 |
| Model 3 | 0.025 (0.008) | <0.01 | -0.005 (0.008) | 0.54 |
| Waist circumference (cm) |  |  |  |  |
| Model 1 | 0.030 (0.006) | <0.001 | 0.013 (0.007) | 0.07 |
| Model 2 | 0.028 (0.006) | <0.001 | 0.009 (0.009) | 0.17 |
| Model 3 | 0.030 (0.007) | <0.001 | 0.001 (0.011) | 0.96 |
| Waist-to-hip ratio |  |  |  |  |
| Model 1 | 0.021 (0.005) | <0.001 | 0.011 (0.006) | 0.06 |
| Model 2 | 0.019 (0.005) | <0.001 | 0.009 (0.006) | 0.13 |
| Model 3 | 0.018 (0.006) | <0.01 | 0.007 (0.006) | 0.20 |
| Waist-to-height ratio |  |  |  |  |
| Model 1 | 0.027 (0.006) | <0.001 | 0.011 (0.006) | 0.07 |
| Model 2 | 0.023 (0.006) | <0.001 | 0.009 (0.006) | 0.11 |
| Model 3 | 0.026 (0.007) | <0.001 | 0.002 (0.006) | 0.74 |
| **15-17 years old** |  |  |  |  |
| Body mass index (kg/m^2^) |  |  |  |  |
| Model 1 | 0.014 (0.008) | 0.10 | 0.010 (0.008) | 0.23 |
| Model 2 | 0.013 (0.010) | 0.17 | 0.012 (0.009) | 0.17 |
| Model 3 | -0.002 (0.013) | 0.85 | -0.007 (0.11) | 0.52 |
| Waist circumference (cm) |  |  |  |  |
| Model 1 | 0.017 (0.008) | 0.04 | 0.012 (0.009) | 0.21 |
| Model 2 | 0.016 (0.009) | 0.09 | 0.014 (0.010) | 0.17 |
| Model 3 | 0.002 (0.011) | 0.89 | 0.001 (0.011) | 0.96 |
| Waist-to-hip ratio |  |  |  |  |
| Model 1 | 0.013 (0.011) | 0.22 | 0.006 (0.010) | 0.53 |
| Model 2 | 0.012 (0.011) | 0.27 | 0.005 (0.010) | 0.64 |
| Model 3 | 0.004 (0.012) | 0.74 | 0.002 (0.010) | 0.84 |
| Waist-to-height ratio |  |  |  |  |
| Model 1 | 0.011 (0.008) | <0.001 | 0.009 (0.008) | 0.28 |
| Model 2 | 0.010 (0.009) | 0.27 | 0.010 (0.009) | 0.26 |
| Model 3 | -0.001 (0.011) | 0.91 | -0.00006 (0.010) | 0.99 |

Model 1: adjusted for age and adulthood family history CVD and smoking status.

Model 2: model 1 + systolic blood pressure, total cholesterol, and fasting blood sugar

Model 3: model 2 + adulthood relevant anthropometric measurement (i.e., for waist circumference, adulthood waist circumference was included in the adjustment model).

P-value < 0.05 is significant

| **Supplementary table 4.** Comparison of the area under receiver operating curves of adolescent anthropometric indices in boys | | | | |
| --- | --- | --- | --- | --- |
|  | Body mass index | Waist circumference | Waist-to-hip ratio | Waist-to-height ratio |
| Body mass index | - | 0.07 | 0.75 | 0.39 |
| Waist circumference | 0.07 | - | 0.75 | 0.07 |
| Waist-to-hip ratio | 0.75 | 0.75 | - | 0.13 |
| Waist-to-height ratio | 0.39 | 0.07 | 0.13 | - |
| Data are shown as p-value | | | | |
